# Phenolic Content of *Hypodaphnis Zenkeri* and Its Antioxidant Effects against Fenton Reactions’ Mediated Oxidative Injuries on Liver Homogenate

**DOI:** 10.3390/antiox3040866

**Published:** 2014-12-16

**Authors:** Bruno Moukette Moukette, Constant Anatole Pieme, Prosper Cabral Nya Biapa, Jacques Romain Njimou, Vicky Jocelyne Ama Moor, Marco Stoller, Marco Bravi, Jeanne Yonkeu Ngogang

**Affiliations:** 1 Laboratory of Biochemistry, Department of Biochemistry and Physiological Sciences; Faculty of Medicine and Biomedical Sciences, University of Yaoundé I, P.O. Box 1364 Yaounde, Cameroon; E-Mails: mouket2006@yahoo.fr (B.M.M.); movicky@yahoo.fr (V.J.A.M.); jngogang@yahoo.fr (J.Y.N.); 2 Laboratory of Medecinal plant Biochemistry, Food Science and Nutrition, Department of Biochemistry, Faculty of Science, University of Dschang, P.O. Box 67 Dschang, Cameroon; E-Mail: brbiapa@yahoo.fr; 3 Department of Chemical Materials Environmental Engineering, Via Eudossiana 18, University of Rome “La Sapienza”, 00185 Rome, Italy; E-Mails: jnjmou@yahoo.fr (J.R.N.); marco.stoller@uniroma1.it (M.S.); marco.bravi@uniroma1.it (M.B.)

**Keywords:** *Hypodaphnis zenkeri*, polyphenols profile, antioxidant, liver homogenate

## Abstract

Under oxidative stress conditions, endogenous antioxidant defenses are unable to completely inactivate the free radicals generated by an excessive production of reactive oxygen species (ROS). This state causes serious cell damage leading to a variety of human diseases. Natural antioxidants can protect cells against oxidative stress. *Hypaodaphnis zenkeri* (*H. zenkiri*) is a plant consumed as a spice in the Cameroonian diet, and its bark has been used in traditional medicine for the treatment of several diseases. The present study aims at investigating the antioxidant activity, which includes free radical scavenging and protective properties of an extract from *H. Zenkiri* against oxidative damage on a liver homogenate. The free radical assays determined the scavenging activities of 2,2-diphenyl-1-picrylhydrazyl (DPPH), hydroxyl (OH), nitrite oxide (NO) and 2,2-azinobis(3-ethylbenzthiazoline)-6-sulfonic acid (ABTS) radicals and the enzymes, whose protection was to be considered in the liver homogenate, including superoxide dismutase, catalase, and peroxidase. The antioxidative activities were studied using the ferric reducing antioxidant power (FRAP), reductive activity, and phosphomolybdenum antioxidant power (PAP) methods. In addition, the phenolic contents of the extracts were examined. The results showed that these extracts demonstrated significant scavenging properties and antioxidant activities, with the hydro-ethanolic extract of the bark of *H. zenkeri* (EEH) being the most potent. This extract had the highest total polyphenol (21.77 ± 0.05 mg caffeic acid (CAE)/g dried extract (DE)) and flavonoids (3.34 ± 0.13 mg quercetin (QE)/g dried extract) content. The same extract had significantly greater protective effects on enzyme activities compared to other extracts. The high performance liquied chromatography (HPLC) profile showed higher levels of caffeic acid, OH-tyrosol acid, and rutin in the leaves compared to the bark of *H. zenkeri*. In conclusion, the ethanolic and hydro-ethanolic extracts of the bark and leaves from *H. zenkeri* showed an antioxidant and protective potential against oxidative damage.

## 1. Introduction

Reactive oxygen species (ROS) are by-products of normal metabolism. They are produced in cells as a response to several factors, including oxidative and thermal stresses, ultraviolet light, chemical agents, and ionizing radiation. These species are known to play a dual role in biological systems, since they can be either harmful or beneficial to living systems [1]. Positive effects of ROS involve physiological roles in cellular responses, such as in defense against infectious agents and in the function of a number of cellular signaling systems. One further beneficial example of ROS at low concentrations is the induction of a mitogenic response [1].

A negative effect is that, in high concentrations, they lead to oxidative stress. This is described as a condition in which cellular antioxidant defenses are inadequate to completely inactivate the free radicals generated by an excessive production of ROS, loss of antioxidant defenses, or both [2]. Oxidative stress can cause DNA damage, cell functions inhibition, lipid and protein peroxidation, and disturbance of glutathione levels. In addition, ROS contribute to the development of cancer, diabetes, atherosclerosis, inflammatory diseases, and ageing [3].

It has been estimated that one human cell is exposed to approximately 1.5 × 10^5^ oxidative hits a day from hydroxyl radicals and other such reactive species [3]. The generation of various free radicals is closely linked with the participation of redox-active metals [4]. The release of iron in the oxidative process is facilitated by ^•^O_2_, and the free ion participates in the Fenton reaction, generating the highly reactive hydroxyl radical [1,5]. Iron-mediated formation of ROS leads to DNA damage and, therefore, appears to be important for the development of cancer, since cancer cells are known to grow rapidly in response to iron [6]. Metal-induced generation of oxygen radicals results in the attack of not only DNA in the cell nucleus but also other cellular components involving polyunsaturated fatty acid residues of phospholipids, which are extremely sensitive to oxidation [7]. Lipid peroxidation is an oxidative deterioration of polyunsaturated lipids, and it involves ROS and transition metal ions, leading to the yielding of a wide range of cytotoxic products, most of which are aldehydes, like malon-dialdehyde (MDA) and 4-hydroxynonrnal (HNE) [8]. The oxidative state created by free radicals also deteriorates proteins with the introduction of carbonyl groups and the formation of protein-centered alkyl groups.

In order to contain the harmful effects of ROS, the body has natural antioxidants that are the cell’s defense mechanisms such as glutathione, alpha-lipoic acid, coenzyme Q, ferritin, uric acid, bilirubin, metallothionein, l-carnitine, melatonin, enzymatic superoxide dismutase (SOD), catalase (CAT), glutathione peroxidases (GPXs), thioredoxins (TRX) and peroxiredoxins (PRXs) which scavenge reactive species [9,10,11,12,13]. Despite the presence of the cell’s antioxidant defense system to counteract oxidative damage from ROS, oxidative damage accumulates during the life cycle and the natural defense could be overcome by the ROS [9]. There is currently immense interest in natural antioxidants and their role in human health and nutrition [14,15,16]. Considerable amount of data has been generated on antioxidant properties of food plants around the globe [12,17,18]. Fruits, vegetables, and herbs attract great attention due to their high content of bioactive compounds with antioxidant characteristics such as phenolic compounds [19,20]. Phenolic compounds are large and heterogeneous groups of secondary plant metabolites that are distributed throughout the plant kingdom. Phenolic compounds include flavonoids, tannins, and phenolic acids [21]. They have been implicated in a number of varied roles including ultra-violet (UV) protection, pigmentation, disease resistance, and nodule production.

*Hypodaphnis zenkeri* is a medium-sized tree of the Lauraceae family found mainly in tropical rain forests in Central and West Africa. *H. zenkiri* is a tree reaching a height of 20 to 25 m [22,23]. Its bark is consumed as a spice in Cameroon and is also used in traditional medicine [23,24]. The aqueous/ethanol extract of the bark of *H. zenkiri* have been postuled to possess quinones, tannins, terpenoids and reducing sugars with a great polyphenolic content and an antioxidant power in the 2,2-azinobis(3-ethylbenzthiazoline)-6-sulfonic acid (ABTS) assay [22,23,24]. A previous study demonstrated that the bark of *H. zenkiri* lowered blood glucose and ameliorated the lipid profile in triton W-1339 induced acute hyperlipidemic rats and rats fed with a high fat and high glucose diet [23]. However the different phenolic compounds are still unknown and the mechanism that is responsible for the antioxidant and the protective effect on hepatic enzymes is not yet elucidated.

The present study aims at determining the phenolic profile of *H. zenkiri* by high performance liquid chromatography (HPLC), investigating the free radical scavenging potential on various free radicals, providing further information on its antioxidant potential and studying its protective effect against oxidative mediated free radical damage on a liver homogenate.

## 2. Material and Methods

### 2.1. Plant Material

The leaves and barks of *H. zenkiri* were collected at the Kala Mountain in the central region of Cameroon. They were authenticated by Nana Pierre, a botanist at the National Herbarium of Cameroon, who compared them to the voucher specimens (16419/SFR/CAM).

### 2.2. Preparation of Plant Extracts

The collected leaves and barks were dried at ambient temperature, crushed, and sifted. The powders were then macerated at the ratio of 1:10 (w/v) for 48 h in ethanol for the ethanolic extract and in a mixture of water/ethanol (30/70; pH = 3) for the hydro-ethanolic extract. The mixtures were then filtered using a Buchner funnel (Thermo Fisher Scientific Inc., Waltham, MA, USA) and Whatman No. 1 filter paper (Whatman International, Maidstone, UK ). This process was repeated once on the residue after 48 h. The supernatant was concentrated using a rotavaporator (Janke & Kunkel, Freiburg, Germany), and the extract was dried in an oven at 55 °C for two days. Each crude extract obtained was labeled using the following codes: EEE: *H. zenkeri (*bark) ethanolic extract; EFE: *H. zenkeri* (leaves) ethanolic extract; EEH: *H. zenkeri* (bark) hydro-ethanolic extract; EFH: *H. zenkeri* (leaves) hydro-ethanolic extract. The different samples were then kept at 4 °C. Prior to the experimentation, the samples of the four plant extracts were reconstituted using the appropriate solvent and different dilutions (25, 50, 75, 150, 300 μg/mL, respectively).

### 2.3. Determination of the Free Radical Scavenging and Antioxidant Properties

#### 2.3.1. Determination of the Free Radical Scavenging Potential of the Samples

##### 2.3.1.1. Scavenging Activity of the 2,2-Diphenyl-1-Picrylhydrazyl (DPPH) Radical

This assay measures the free radical scavenging capacity of the investigated extracts [25]. Briefly, in 3 mL of each diluted extract or vitamin C used as standard, 1 mL of methanol solution of DPPH 0.1 mM was added. The mixture was kept in the dark at room temperature for 30 min and the absorbance was measured at 517 nm against a blank. The following equation was used to determine the percentage of the radical scavenging activity of each extract.

(1) $$Scavenging\;effect\;\left(\%\right)=100\;\times \left(A_{o}-A_{s}\right)/A_{o}$$

where *A*_o_ is the absorbance of the blank and *A*_s_ the absorbance of the sample.

##### 2.3.1.2. Scavenging Effect of the ABTS^+^ Radical

The ABTS assay was based on a previously described method [26] with slight modifications. The ABTS radical cation (ABTS^+^) was produced by the reaction of a 7 mM ABTS solution with 2.45 mM potassium persulfate. The mixture was stored in the dark at room temperature for 12 h before use. The ABTS^+^ solution was diluted with ethanol to obtain an absorbance of 0.70 ± 0.05 at 734 nm. After addition of 25 μL of extract samples or vitamin C used as standard to 2 mL of diluted ABTS^+^ solution, absorbance was measured at 734 nm after exactly 6 min. The decrease in absorption was used for calculating scavenging effect values. The following equation was used to determine the percentage of the radical scavenging activity of each extract.

(2) $$Scavenging\;effect\;\left(\%\right)=100\;\times \left(A_{o}-A_{s}\right)/A_{o}$$

where *A*_o_ is the absorbance of the blank; *A*_s_ is the absorbance of the sample.

##### 2.3.1.3. Nitric Oxide Scavenging Activity

Nitric oxide scavenging activity was determined according to the Griess Illosvoy reaction [27]. The reaction mixture contained 2 mL of 10 mM SNP (sodium nitro-prusside) in 0.5 mL of phosphate buffer (0.5 M; pH 7.4). Various concentrations (25, 50, 75, 150, 300 μg/mL) of the extracts (0.5 mL) were added in a final volume of 3 mL. After incubation for 60 min at 37 °C, Griess reagent (0.1% α-napthyl-ethylenediamine in water and 1% sulfanilic acid in 5% H_3_PO_4_) was added. The pink chromophore generated during diazotization of nitrite ions with sulfanilamide and subsequent coupling with α-napthyl-ethylenediamine was measured spectrophotometrically at 540 nm. Ascorbic acid was used as a positive control. Nitric oxide scavenging ability (%) was calculated using the formula:

(3) $$\text{Percentage of NO radical scavenging activity }\left(\%\right)=100\;\times \left(A_{o}-A_{s}\right)/A_{o}$$

where *A*_o_ is the absorbance of the blank and *A*_s_ the absorbance of the sample.

##### 2.3.1.4. Hydroxyl Radical Scavenging Activity

The scavenging activity of the extracts on the hydroxyl radical was measured according to a previously described method [28]. In 1.5 mL of each diluted extract, 60 μL of FeCl_3_ (1 mM), 90 μL of 1,10-Phenanthroline (1 mM), 2.4 mL of 0.2 M phosphate buffer, (pH 7.8) and 150 μL of H_2_O_2_ (0.17 M) were added, respectively. The mixture was then homogenized and incubated at room temperature for 5 min. The absorbance was read at 560 nm against the blank. The percentage of the radical scavenging activity of each extract was calculated from the equation below:

(4) $$\text{Percentage of OH radical scavenging activity }\left(\%\right)=100\;\times \left(A_{o}-A_{s}\right)/A_{o}$$

where *A*_o_ is the absorbance of the blank and *A*_s_ the absorbance of the sample.

#### 2.3.2. Determination of the Total Antioxidant Potential of the Different Samples

##### 2.3.2.1. Total Antioxidant Activity by Ferric Reducing Antioxidant Power Assay (FRAP)

The FRAP was determined using a previously described method [29] with slight modifications. The fresh FRAP reagent consisted of 500 mL of acetate buffer (300 mM pH 3, 6), 50 mL of 2,4,6-Tri (2-pyridyl)-*s*-triazin (TPTZ) (10 mM), and 50 mL of FeCl_3_·6H_2_O (50 mM). The colorimetric measurement was performed at 593 nm, and the reaction was monitored up to 12 min using the mixture of 75 μL of each extract and 2 mL of FRAP reagent. The ascorbic acid was used to draw a standard curve and the butylated hydroxyl toluene (BHT) was used for the comparison. The absorbance was read at 593 nm. The result where expressed as mg equivalent vitamin C/g of dried extract (mg eq Vit C/g DE).

##### 2.3.2.2. Phosphomolybdenum Antioxidant Power (PAP)

The total antioxidant activity of extracts was evaluated by green phosphomolybdenum complex according to a described method [30]. An aliquot of 10 μL of extract samples solution was mixed with 900 μL of reagent solution (0.6 M sulphuric acid, 28 mM sodium phosphate and 4 mM ammonium molybdate) in a micro centrifuge tube for a final volume of 1 mL. Tubes were incubated in a dry thermal bath at 95 °C for 90 min. After cooling, the absorbance of the mixture was measured at 695 nm against a blank. Ascorbic acid was used as reference to draw the standard curve and BHT was used as comparison. The reducing capacities of the analyzed extracts were expressed as mg of ascorbic acid (AS) equivalents/g of dried extract (mg eq AS/g DE).

##### 2.3.2.3. Reducing Power Assay

The reducing power of the extracts was determined by a method described by Oyaizu (1996) [31]. Different concentrations (25, 50, 75, 150, 300 μg/mL) of extracts in 1 mL of distilled water were mixed with 2.5 mL of phosphate buffer (0.2 M, pH 6.6) and 2.5 mL of potassium ferrocyanide (1%). The mixture was incubated at 50 °C for 20 min. Aliquots (2.5 mL) of trichloroacetic acid (10%) were added to the mixture and centrifuged at 3000 rpm for 10 min. The upper layer of the solution (2.5 mL) was mixed with 2.5 mL of distilled water and 0.5 mL of FeCl_3_ (0.1%). Increasing absorbance was measured at 700 nm against the blank indicates increased reducing power.

#### 2.3.3. Determination of the Phenolic Content of Extracts

##### 2.3.3.1. Total Phenol Determination

The total phenol was determined by the Folin–Ciocalteu method [32], the reaction mixture contained 200 μL of diluted extracts, 800 μL of freshly prepared diluted Folin Ciocalteu reagent and 2 mL of 7.5% sodium carbonate. The final mixture was diluted to 7 mL with deionized water and kept in the dark at ambient conditions for 2 h to complete the reaction. The absorbance at 765 nm was measured. Caffeic acid was used as standard, and the results were expressed as mg caffeic acid/g dried extract (mg CAE/g DE).

##### 2.3.3.2. Determination of Total Flavonoid Content

Total flavonoid content was determined using aluminum chloride (AlCl_3_) according to a known method [33] using quercetin as a standard. Extract (0.1 mL) was added to 0.3 mL distilled water followed by 0.03 mL of NaNO_2_ (5%). After incubation of 5 min at 25 °C, 0.03 mL of AlCl_3_ (10%) was added. After a further 5 min, the reaction mixture was treated with 0.2 mL of 1 mM NaOH. Finally, the reaction mixture was diluted to 1 mL with water and the absorbance was measured at 510 nm. The results were expressed as mg quercetin (QE) of dried extract (mg QE/g DE).

##### 2.3.3.3. Determination of Total Flavonols

Total flavonols in the plant extracts were estimated using a known method [34] with modifications. To 2.0 mL of sample or standard, 2.0 mL of AlCl_3_ (2%) and 3.0 mL of sodium acetate solution (50 g/L) were added. The mixture was incubated for 2.5 h at 20 °C and the absorption was read at 440 nm. Extract samples were evaluated at a final concentration of 0.1 mg/mL. Total flavonols content was calculated as quercetin (mg/g) using the equation *y* = 5.3911 *x* + 0.0313; *R*^2^ = 0.9967, where x is the absorbance and y, the concentration of quercetin equivalent of dried extract (mg QE/g DE).

#### 2.3.4. Quantification of Phenolics by HPLC

High Performance Liquid Chromatography (HPLC) with ultra violet (UV) detection is frequently used for the separation and detection of phenolic compounds present in extracts. Samples were dissolved in pure water according to the ratio (0.3 g/10mL) and centrifuged at 4706 rpm for 10 min. The supernatant was filtered through a cellulose acetate membrane filter (0.20 μm or 0.45 μm, Schleicher & Schuell, Roma, Italy) and used for analysis. A portion of 25 μL of the filtrate was injected into the HPLC system and eluted as described below. The analysis was performed on an Agilent Technologies 1200 HPLC system fitted with a SUPELCOSIL LC-18 column (length 250 mm, diameter 4.6 mm, packaging size 5 mm, (TK mediterranea™ Sea 18, Roma, Italy). The column temperature was set at 20 °C. The mobile phase consisted of a mixture of an aqueous solution of acetic acid at 0.5% by volume (A) and acetic nitrile (B). Elution was performed by according to the following protocol:

- At start and for the first 2 min of the run, 100% of A.
- From 2 min to 60 min after the run start, a linear composition ramp was used, targeting 40% of A and 60% of B.

The flow rate was set equal to 1 mL/min. Polyphenols were detected by a UV detector (280 nm, TK mediterranea™ Sea 18, Roma, Italy). Beforehand, the retention times of the identified polyphenolic compounds of interest available were measured by using of single standard solution at a concentration of 100 mg/L.

#### 2.3.5. Protective Properties of the Plant against Oxidative Damage

##### 2.3.5.1. Preparation of Liver Homogenate

The liver was isolated from three normal albino *Wistar* rats. The organs were weighed and 10% (w/v), the homogenate was prepared in phosphate buffer (0.1 M, pH 7.4 having 0.15 M KCl) using the homogenizer at 4 °C [35]. The homogenate was centrifuged at 3000 rpm for 15 min and the clear cell-free supernatant obtained was used for the study.

##### 2.3.5.2. Preparation of the Pro-Oxidative Solution

The oxidant solution was prepared immediately before its utilization by adding a solution of ferric chloride 100 mM to H_2_O_2_ 0.50% prepared in phosphate buffer (0.1 M, pH 7.4). This solution was used for the investigation of the protective assays on liver enzymes.

##### 2.3.5.3. Total Protein Content

The total protein content of the mixture of liver was measured according to the protein kit supplier method (Human Kit-Hu102536, Humann, Wiesbaden, Germany). This result was used to express the activities of the different enzymes per gram of organs.

##### 2.3.5.4. *In Vitro* Lipid Peroxidation Assay

Lipid peroxidation assay was performed by a formerly described protocol [10]. Phosphate buffer 0.58 mL (0.1 M; pH 7.4), 200 μL sample, 200 μL liver homogenate, and 20 μL ferric chloride (100 mM) were combined to form a mixture, which was placed in a shaking water bath for 1 h at 37 °C. The reaction was terminated by adding 1 mL trichloroacetic acid (10%), 2-thiobarbituric acid 1 mL (0.67%) to all the tubes, which were placed in a boiling water bath for 20 min. Then the test tubes were shifted to a crushed ice bath and were centrifuged at 3000 rpm for 10 min. Absorbance of the supernatant was checked at 535 nm and was calculated as nM of MDA tissue using a molar extinction coefficient of 1.56 × 10^5^/M.cm.

##### 2.3.5.5. Determination of Peroxidase Activity

Peroxidase activity was determined by the peroxidase kit (CAS Number 7722-84-1, Munich, Germany) supplier with modifications. A solution containing the mixture of 1 mL of the oxidant solution (FeCl_3_, 100 mM) and extract or vitamin C (standard) for a final concentration of 100 μg/mL was incubated for 1 h in a water bath at 37 °C. An aliquot of phosphase buffered saline (PBS) (0.1 mL), hydrogen peroxide (50 μL), and pyrogallol solution (110 μL) were added to distilled water (625 μL) that had been dispensed into an Eppendorf tube (Fisher Scientific, Wohlen, Switzerland). The plant extract (75 μL) from the mixture was thereafter added. For the blank, the control oxidant solution and the vitamin C as standard, the same reagents were used, except the extract, which was replaced by distilled water (75 μL). The reaction was mixed and incubated for at least 10 min. The solution containing 100 mM, pH 6.0 PBS (40 μL) and 0.002% (v/v) diluted liver homogenate (40 μL) was added to the blank and test mixtures, respectively. These were mixed, and the increase in absorbance at 420 nm was measured every 10 s for 3 min using a spectrophotometer (BioMate 3S UV-Visible, Thermo Scientific™, Wohlen, Switzerland). One unit of peroxidase was defined as the change in absorbance/seconds/mg of protein at 420 nm using molar extinction coefficient of 12/M.cm.

##### 2.3.5.6. Determination of Catalase Activity

Prior to the test, a solution containing a mixture of 1 mL of total volume of the oxidant solution and extract or vitamin C (standard) for a final concentration of 100 μg/mL was incubated for 1 h in a water bath at 37 °C. The catalase activity of liver homogenate was assayed as previously described with modifications [10]. An aliquot of hydrogen peroxide (0.8 mL) was dispensed into an Eppendorf tube. Phosphate buffer (1.0 mL), extracted sample/vitamin C/oxidant solution (75 μL) and (0.002% v/v) diluted homogenate (125 μL) were added. The reaction mixture (0.5 mL) was dispensed into 5% dichromate reagent (1.0 mL) and vigorously shaken. The mixture was heated in a Clifton water bath for 10 min, and allowed to cool. The absorbance at 570 nm was taken using spectrophotometer (BioMate 3S UV-Visible, Thermo Scientific^TM^, Wohlen, Switzerland). The absorbance obtained was extrapolated from the following standard curve: *y* = 0.0028 *x* + 0.0132. The catalase activity was thereafter expressed as Unit/min/mg of protein (UI/mg Prot.).

(5) CAT(unit/mg protein)=(Abs/min×30000 units)/(40cm/M×mg protein)×df

where *df* = dilution factor, *Abs* = absorbance.

##### 2.3.5.7. Superoxide Dismutase (SOD) Activity

The measurement of total SOD activity was performed according to the Misra and Fridovich method [36] with some slight modifications. The principle of this method is based on the inhibition of epinephrine autoxidation. Distilled water (0.2 mL) and 2.5 mL sodium carbonate buffer 0.05 M, pH 10.2 were added to the 0.3 mL buffered epinephrine to initiate the reaction. The absorbance at 480 nm was read for 150 s at 30 s intervals against a blank made up of 2.5 mL buffer, 0.3mL epinephrine and 0.2 mL distilled water. The following equation allowed the calculation of the SOD activity:

(6) SOD(unit/mg protein)=SOD(units/mL/min)/protein(mg/mL )×df

where *df* = dilution factor. The SOD activity was there after expressed as Unit/min/mg of protein (UI/mg Prot.).

#### 2.3.6. Statistical Analysis

The results were presented as mean ± SEM of triplicate assays. Analyses of data were conducted using one-way ANOVA (Analysis of variance) followed by the Kruskal Wallis test and Dunnett’s multiple test (SPSS program version 18.0 for Windows, IBM Corporation, New York, NY, USA). The Log probit was used to determinate the IC_50_ using the software XLstat version 7 (Addinsoft, New York, NY, USA) were used to achieve the Pearson Correlation Analysis (PCA). The differences were considered as significant at *p* < 0.05.

## 3. Results and Discussion

**Figure 1:**
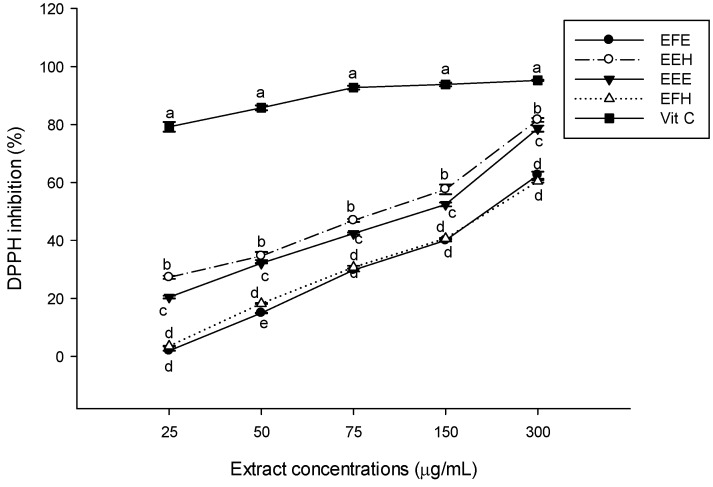
2,2-diphenyl-1-picrylhydrazyl (DPPH) scavenging potential of the different plant extracts. Values are expressed as mean ± standard deviation (SD) of three replicates. In the same concentration the values affected with different letter are significantly different at *p* < 0.05. EEE: *H. zenkeri (*bark) ethanolic extract; EFE: *H. zenkeri* (leaves) ethanolic extract; EEH: *H. zenkeri* (bark) hydro-ethanolic extract; EFH: *H. zenkeri* (leaves) hydro-ethanolic extract; Vit C: Vitamin C.

**Figure 2:**
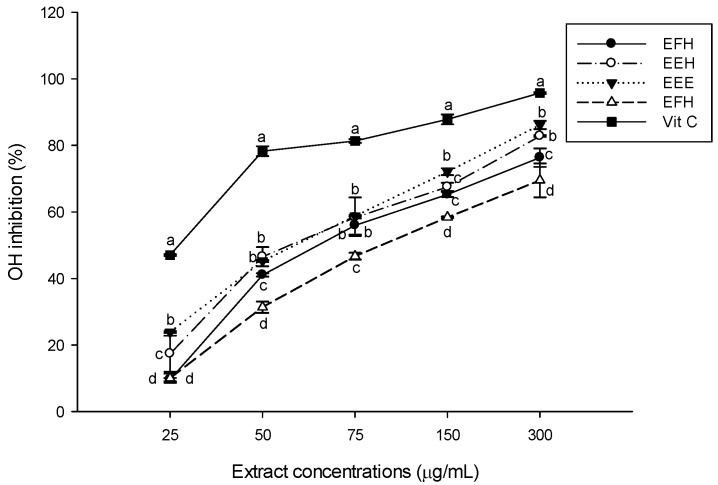
Hydroxyl radical (OH) scavenging potential of the different plant extracts. Values are expressed as mean ± SD of three replicates. In the same concentration the values affected with different letter are significantly different at *p* < 0.05. EEE: *H. zenkeri (*bark) ethanolic extract; EFE: *H. zenkeri* (leaves) ethanolic extract; EEH: *H. zenkeri* (bark) hydro-ethanolic extract; EFH: *H. zenkeri* (leaves) hydro-ethanolic extract; Vit C: Vitamin C.

In order to reduce the oxidative damage induced by free radicals in biological systems, several dietary sources of antioxidants have been investigated. Phytochemicals such as phenolic compounds and some amino acids, have been found to have outstanding antioxidant properties against some free radical species [37]. Regarding the increase of interest in natural antioxidant supplements from foods, medicinal plants, and spices, this study was designed to investigate the antioxidant properties of *H. zenkiri* used in Cameroon as a spice in several dishes. Antioxidant activity cannot be measured directly but rather by the effects of the antioxidant in controlling the extent of oxidation [38]. A rapid, simple, and inexpensive method to measure antioxidant capacity of food involves the use of the free radical 2,2-diphenyl-1-picrylhydrazyl (DPPH). The results of the scavenging activity using DPPH has shown that EEH extract has the highest and most significant (*p* < 0.05) scavenging potential among the extracts with a percentage of inhibition of 27.27% ± 0.45% at the concentration of 25 μg/mL to 81.61% ± 0.66% at 300 μg/mL followed by EEE (Figure 1). However, this result is lower than the activity vitamin C, which was used as control. EFE and EFH showed the lowest scavenging powers at all the concentrations. Our results corroborate previous findings that stated that phenolic compounds generally exhibited significant scavenging effects against the DPPH free radical [9,38]. DPPH is widely used to test the ability of compounds to act as free radical scavengers or hydrogen donors. It has been used to quantify the antioxidants in complex biological systems. The DPPH method can be used for solid or liquid samples, and it is not specific to any particular antioxidant component, but applies to the overall antioxidant capacity of the sample. A measure of total antioxidant capacity helps in the understanding of functional properties of foods [13]. The hydroxyl radical scavenging potential of the different extracts displayed in Figure 2 demonstrated that all the tested extracts scavenged the OH radical with variable scavenging powers depending on the type of extract and the concentration. The inhibitory properties of EEE and EEH extracts increased from 23.86% ± 0.28% to 86.61% ± 1.25% and from 17.37% ± 5.42% to 82.61% ± 0.28%, respectively, demonstrating that they have almost similar and the highest scavenging power of the substances tested. Vitamin C showed the significant < highest (*p* < 0.05) overall potential. Our results show that these plant extracts have the ability to inhibit the non-specific hydroxyl radical, preventing it from reacting with polyunsaturated fatty acid moieties of cell membrane phospholipids and thereby causing damage to cells [39].

**Figure 3:**
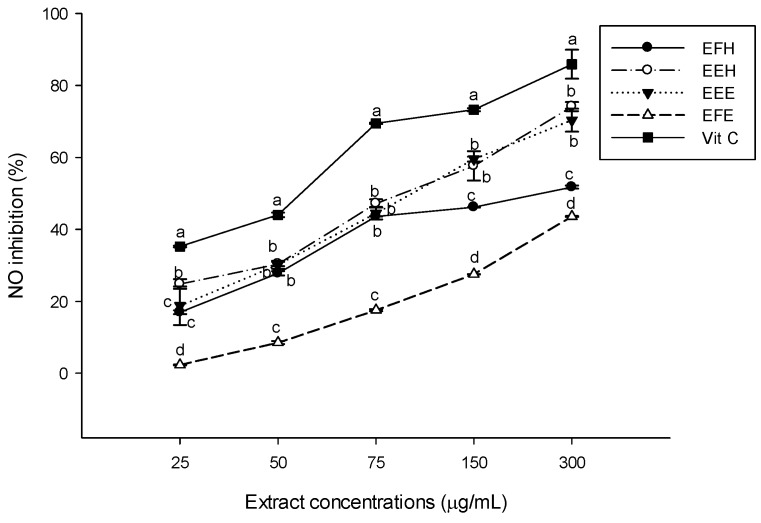
Nitric oxide radical (NO) scavenging potential of the different plant extracts. Values are expressed as mean ± SD of three replicates. In the same concentration the values labeled with different letters differ significantly at *p* < 0.05. EEE: *H. zenkeri* (bark) ethanolic extract; EFE: *H. zenkeri* (leaves) ethanolic extract; EEH: *H. zenkeri* (bark) hydro-ethanolic extract; EFH: *H. zenkeri* (leaves) hydro-ethanolic extract; Vit C: Vitamin C.

The nitric oxide (NO) scavenging assay measures the ability of the extract to chelate NO using the Griess reaction. The nitrite ions were generated from sodium nitroprusside in aqueous solution at a physiological pH, which reacted with oxygen [39]. The different extracts scavenged the NO radical in a concentration-dependent manner (Figure 3). Among the tested extracts, EEE and EEH were the significantly (*p* < 0.05) most potent at all the concentrations but their NO scavenging capacities were lower than that of vitamin C. The lowest scavenging activity was shown by EFE. EFE therefore demonstrates the ability of *H zenkiri* to be a potential free radical quencher.

**Figure 4:**
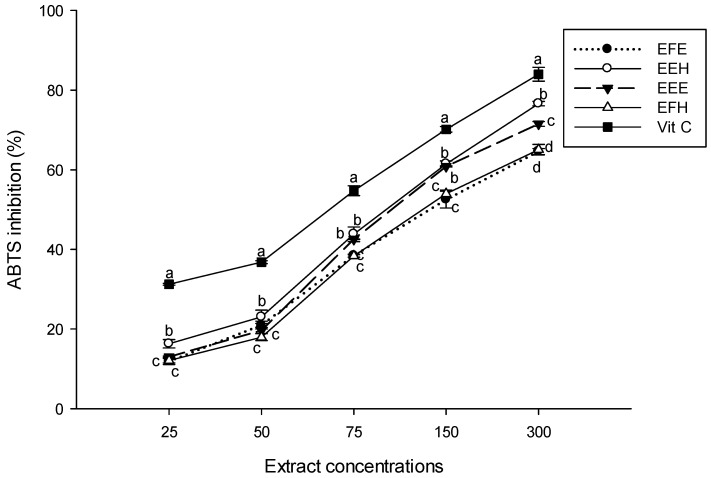
The 2,2-azinobis(3-ethylbenzthiazoline)-6-sulfonic acid (ABTS) scavenging potential of the different plant extracts. Values are expressed as mean ± SD of three replicates. In the same concentration the values labeled with different letters differ significantly at *p* < 0.05. EEE: *H. zenkeri (*bark) ethanolic extract; EFE: *H. zenkeri* (leaves) ethanolic extract; EEH: *H. zenkeri* (bark) hydro-ethanolic extract; EFH: *H. zenkeri* (leaves) hydro-ethanolic extract; Vit C: Vitamin C.

The ABTS method has the extra flexibility in that it can be used at different pH levels and is thus useful when studying the effect of pH on antioxidant activity of various compounds. It is also useful for measuring antioxidant activity of samples extracted in acidic solvents [13]. The inhibitory potential on the ABTS radical of the extracts showed that all the tested samples inhibited the ABTS radical, and the inhibition percentage increased with concentration (Figure 4). The extract EEH had a significantly (*p* < 0.05) more powerful inhibitory potential while the EFH and EFE extracts had the lowest scavenging potentials at all the concentrations. This result suggests that our extracted phytochemicals could act as radical scavengers owing to their hydrogen and electron donating capacity and their ability to delocalize/stabilize the resulting phenoxyl radical within the structure [40].

**Figure 5:**
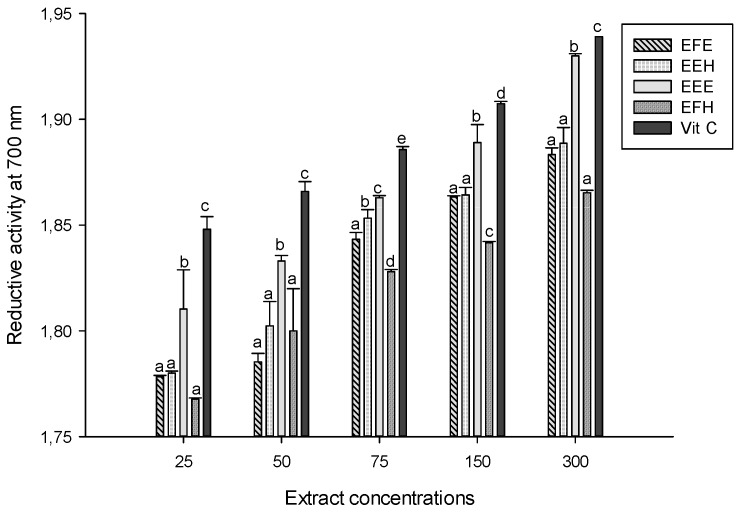
Reductive activity of the different plant extracts. Values are expressed as mean ± SD of three replicates. In the same concentration the values labeled with different letters differ significantly at *p* < 0.05. EEE: *H. zenkeri (*bark) ethanolic extract; EFE: *H. zenkeri* (leaves) ethanolic extract; EEH: *H. zenkeri* (bark) hydro-ethanolic extract; EFH: *H. zenkeri* (leaves) hydro-ethanolic extract; Vit C: Vitamin C.

The reducing capacity of extracts may serve as an indicator of their potential antioxidant activity. Higher absorbance indicates a higher reducing power and higher antioxidant activity [37]. The results of the reductive power of the different extracts revealed that the extract EEE had the highest activity of all extracts (Figure 5). The other extracts exhibited poor activity and were not significantly (*p* < 0.05) different at the concentrations that were used in this study. Vitamin C that was used as standard showed the highest activity at all the concentrations. This activity could be attributed to phenolic compounds that have been identified in these extracts.

**Figure 6:**
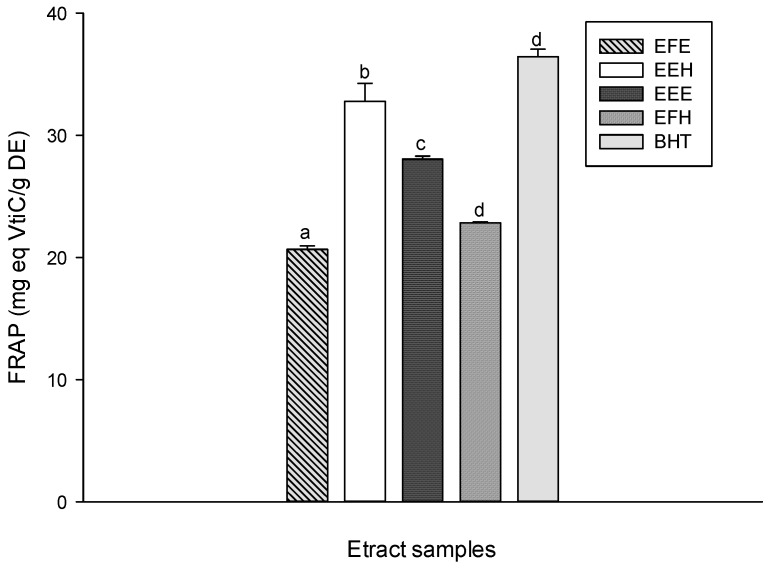
Ferric reducing antioxidant power (FRAP) antioxidant activities of the different plant extracts. Values are expressed as mean ± SD of three replicates. The values labeled with different letters differ significantly different at *p* < 0.05. EEE: *H. zenkeri (*bark) ethanolic extract; EFE: *H. zenkeri* (leaves) ethanolic extract; EEH: *H. zenkeri* (bark) hydro-ethanolic extract; EFH: *H. zenkeri* (leaves) hydro-ethanolic extract; BHT: butylated hydroxyl toluene.

The ferric reducing antioxidant power (FRAP) method is based on the reduction of a ferroin analog, the Fe^3+^ complex of tripyridyltriazine Fe(TPTZ)^3+^, to the intensely blue colored Fe^2+^ complex Fe(TPTZ)^2+^ by antioxidants in acidic medium. The resulting FRAP activity is shown by the increase of the absorbance at 593 nm [38]. The FRAP anti-oxidative power of the different tested extracts is represented in Figure 6. These results show that EEH has the significantly (*p* < 0.05) highest antioxidant power among the tested extracts (32.79 ± 1.46 mg eq Vit C/g DE) followed by EEE (28.05 ± 0.24 mg eq Vit C/g DE). The amount of Fe^3+^ ions reduced to Fe^2+^ ions varied with the concentration of phenols. The BHT used as standard had the highest and most significant (*p* < 0.05) FRAP antioxidant power.

**Figure 7:**
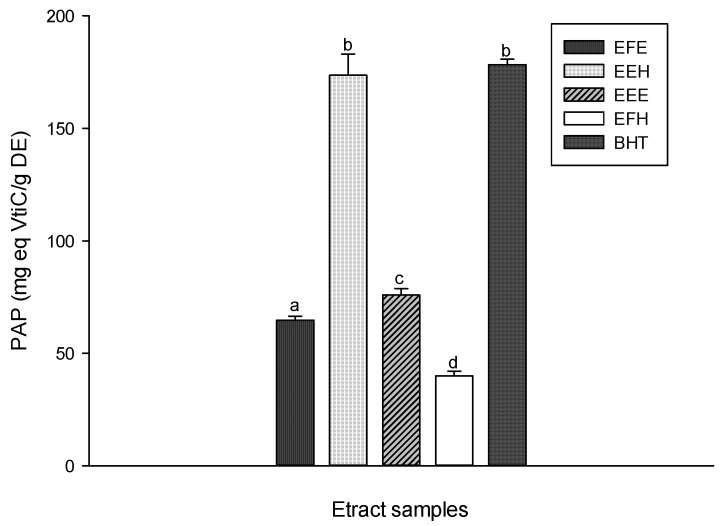
Phosphomolybdenum antioxidative power (PAP) of the different plant extracts. Values are expressed as mean ± SD of three replicates. The values labeled with different letters differ significantly at *p* < 0.05. EEE: *H. zenkeri (*bark) ethanolic extract; EFE: *H. zenkeri* (leaves) ethanolic extract; EEH: *H. zenkeri* (bark) hydro-ethanolic extract; EFH: *H. zenkeri* (leaves) hydro-ethanolic extract; BHT: butylated hydroxyl toluene.

The results of the phosphomolybdenum antioxidative power (PAP) of the different samples presented in Figure 7 showed that all the tested extracts had a PAP and that this property varied with the type of extract. The EEH extract showed the higher PAP among the extracts (173.58 ± 9.37 mg eq Vit C/g DE). The above results are similar to that of the BHT (178.26 ± 2.42 mg eq Vit C/g DE). These results support the hypothesis that our extract could act also as ion chelator.

Phenolic compounds are involved in repair and adaptive systems and can act preventively or therapeutically in various diseases [40,41]. Table 1 presents the phenolic composition of the different extracts. These results show that the extract EEH (21.77 ± 0.05 caffeic acid (CAE)/g DE) has the highest total phenol concentration followed by EEE (18.38 ± 0.09 CAE/g DE). Similar results were noted for flavonoids and flavonols. These results underline the high antioxidant activities observed in EEH and EEE extracts. There is a strong relationship between total phenolic and flavonoid contents and antioxidant, free radical scavenging activities in many plants although this depends on their proportions in the plants [40].

**Table 1:** Total phenol, flavonoid, and flavonol contents of the different plant extracts.

Samples	Phytochemicals		
	Total Phenols (mg CAE/g DE)	Flavonoids (mg QE/g DE)	Flavonols (mg CAE/g DE)
EEE	18.38 ± 0.09 ^a^	2.58 ± 0.57 ^a^	1.10 ± 0.41 ^a^
EEH	21.77 ± 0.05 ^b^	3.34 ± 0.13 ^a^	1.47 ± 0.03 ^a^
EFH	10.70 ± 0.01 ^c^	1.40 ± 0.35 ^b^	0.71 ± 0.06 ^b^
EFE	7.21 ± 0.05 ^d^	0.50 ± 0.18 ^c^	0.27 ± 0.01 ^c^

Values are expressed as mean ± SD of three replicates. The values labeled with different letters differ significantly at *p* < 0.05. EEE: *H. zenkeri (*bark) ethanolic extract; EFE: *H. zenkeri* (leaves) ethanolic extract; EEH: *H. zenkeri* (bark) hydro-ethanolic extract; EFH: *H. zenkeri* (leaves) hydro-ethanolic extract. DE: dried extract; QE: quercetin; CAE; caffeic acid.

**Figure 8:**
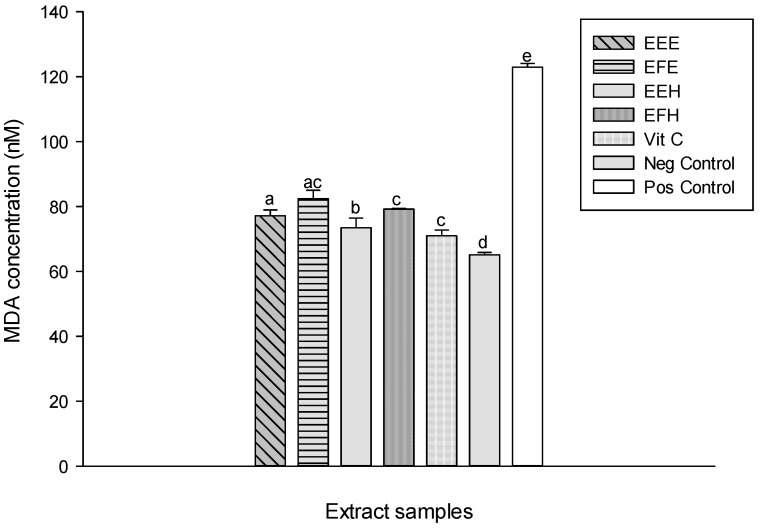
Protective properties of plant extracts against lipid peroxidation. Values are expressed as mean ± SD of three replicates. In the same column the values labeled with different letters differ significantly at *p* < 0.05. EEE: *H. zenkeri (*bark) ethanolic extract; EFE: *H. zenkeri* (leaves) ethanolic extract; EEH: *H. zenkeri* (bark) hydro-ethanolic extract; EFH: *H. zenkeri* (leaves) hydro-ethanolic extract; Vit C: Vitamin C. Pos Control: oxidant (positive) control. Neg Control: Normal (negative) control.

**Figure 9:**
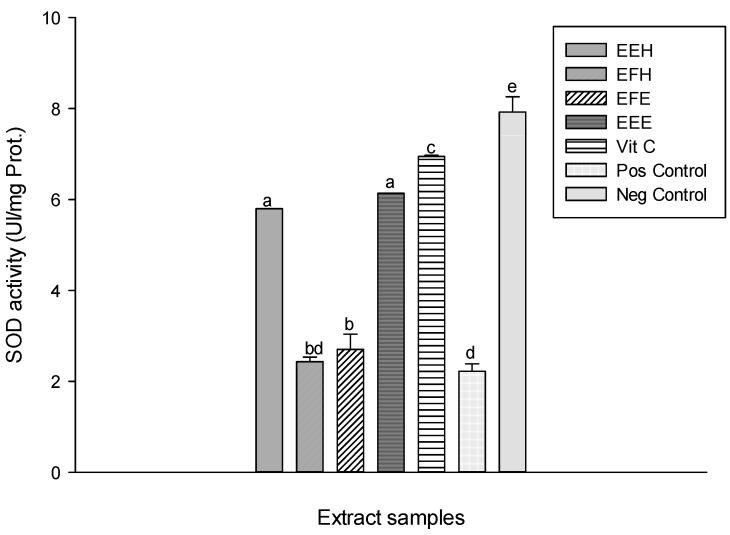
Protective properties of plant extracts: SOD activity. Values are expressed as mean ± SD of three replicates. In the same column the values labeled with different letters differ significantly at *p* < 0.05. EEE: *H. zenkeri (*bark) ethanolic extract; EFE: *H. zenkeri* (leaves) ethanolic extract; EEH: *H. zenkeri* (bark) hydro-ethanolic extract; EFH: *H. zenkeri* (leaves) hydro-ethanolic extract; Vit C: Vitamin C. Pos Control: oxidant (positive) control. Neg Control: Normal (negative) control.

**Figure 10:**
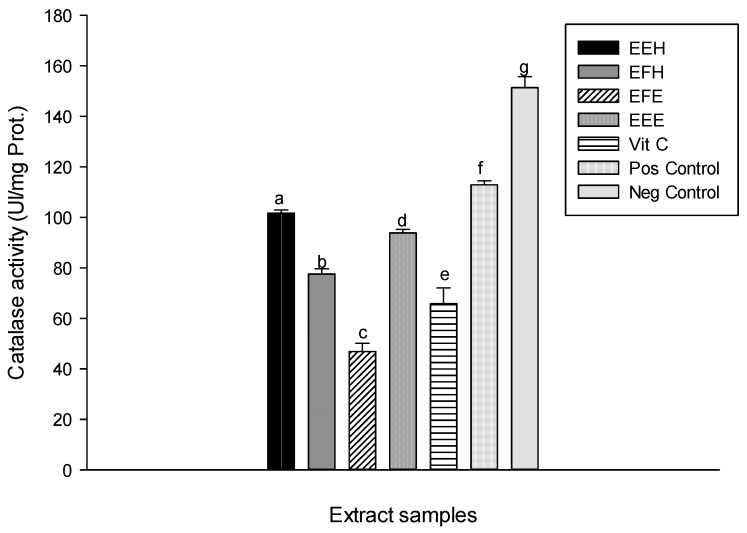
Protective properties of plant extracts: catalase activity. Values are expressed as mean ± SD of three replicates. In the same column the values labeled with different letters differ significantly at *p* < 0.05. EEE: *H. zenkeri (*bark) ethanolic extract; EFE: *H. zenkeri* (leaves) ethanolic extract; EEH: *H. zenkeri* (bark) hydro-ethanolic extract; EFH: *H. zenkeri* (leaves) hydro-ethanolic extract; Vit C: Vitamin C. Pos Control: oxidant (positive) control. Neg Control: Normal (negative) control.

Lipid peroxidation is one of the major ways by which ROS cause cell damage since peroxidation results in changes in fluidity, membrane permeability, and alteration of functions of proteins that are embedded in the membrane [42]. Natural products from medicinal plants can reduce this deleterious process [10]. The protective effects of the extracts tested against lipid peroxidation are presented in Figure 8. This result shows a significant (*p* < 0.05) increase of the oxidant level (positive) (122.95 ± 2.42 nM) compared to the normal (negative) (65.13 ± 0.72 nM). The different tested extracts have shown the reduction in MDA level. The extract EEH has the most significant and highest inhibition (73.21 ± 3.02 nM) as demonstrated by its lowest MDA level. This result suggests that the polyphenol compounds present in the extract may either delay or inhibit the initiation step of lipid peroxidation by reacting with a lipid radical or by inhibiting their propagation step or by reacting with peroxyl or alkoxyl radicals [38,43]. Also, the antioxidants from the extracts may further interfere with chain propagation reactions by forming peroxy-antioxidant stable compounds [38]. ROS damage macromolecules in cells and have been involved in the etiologies or manifestations of several pathological processes [2,44]. Antioxidant enzymes are capable of eliminating ROS and lipid peroxidation products, therefore, they can protect cells and tissues from oxidative damage. Antioxidant enzymes include SOD, catalase and peroxidases. SOD is a key enzyme in the natural defense against free radicals, and its determinant role has been described [45]. It is well known that the superoxide ion (O_2_^−^) is the starting point in the chain production of free radicals. At this early stage, superoxide dismutase inactivates the superoxide ion by transforming it into hydrogen peroxide (H_2_O_2_) [45]. The SOD activity of the different tested samples showed an increase of the SOD activity of the different extracts compared to the oxidant 2.22 ± 0.16 UI/mg Prot. (Figure 9). The extracts EEE and EEH showed the highest activities with values of 5.79 ± 0.02 UI/mg Prot and 6.13 ± 0.03 UI/mg Prot., respectively). This result suggests that our extracts contain molecules that enable them to act as secondary or preventative antioxidants that retard the rate of oxidation. These extracts may achieve this activity through several ways including the removal of substrate or singlet oxygen quenching therefore protecting the enzymes [38]. Hydrogen peroxide (H_2_O_2_), a highly ROS, is produced as the result of SOD reactions [46]. The catalase activity recorded in the different groups is displayed in Figure 10. This result shows a significant (*p* < 0.05) decrease of the catalase activity in the oxidant (positive) (5.22 ± 0.92 UI/mg Prot.) compared to the negative (40.88 ± 2.66 UI/mg Prot.). This activity increases in the different extracts tested with the EEH extract showing the significant and higher activity (101.67 ± 0.92 UI/mg Prot.). The catalases provide protection against the toxic effects of H_2_O_2_ that is generated in respiring cells due to its catalytic function [44,47].

**Figure 11:**
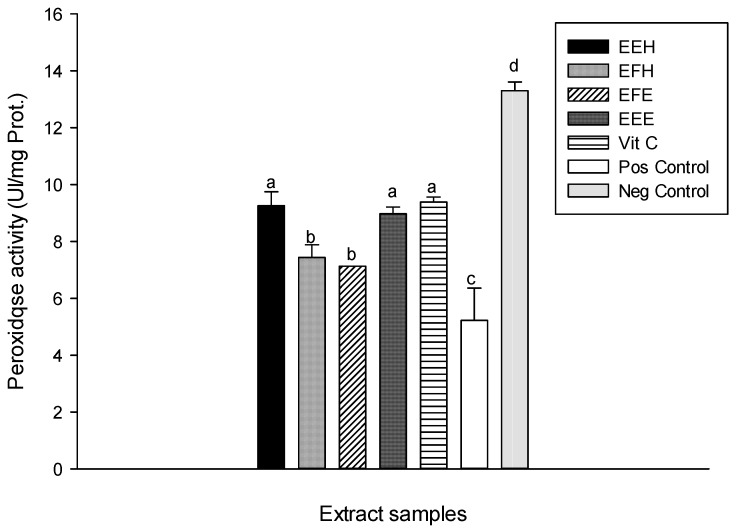
Protective properties of plant extracts: peroxidase activity. Values are expressed as mean ± SD of three replicates. In the same column the values labeled with different letters differ significantly at *p* < 0.05. EEE: *H. zenkeri (*bark) ethanolic extract; EFE: *H. zenkeri* (leaves) ethanolic extract; EEH: *H. zenkeri* (bark) hydro-ethanolic extract; EFH: *H. zenkeri* (leaves) hydro-ethanolic extract; Vit C: Vitamin C. Pos Control: oxidant (positive) control. Neg Control: Normal (negative) control.

This protective potential of plant extracts against enzyme depletion has been attributed to phenolic compounds [41]. Functionally, catalases are related to peroxidases which both degrade H_2_O_2_ to O_2_ and H_2_O [44]. The protective effect of the different plant extracts on peroxidase activity is presented in Figure 11. This result shows the increase of the peroxidase activity of different extracts compared to the oxidant (positive) control. Among the tested samples, the extracts EEH (9.25 ± 0.40 UI/mg Prot.) and EEE (8.97 ± 0.19 UI/mg Prot.) show similar results to vitamin C (9.38 ± 0.14 UI/mg Prot.). This result confirms that phenolic compounds can act as ion chelators and can, therefore, prevent the involvement of hydroxyl radical in Fenton reactions, which can generate higher concentrations of hydroxyl radicals that could in turn injure macromolecules such as enzymes [10,40].

**Figure 12:**
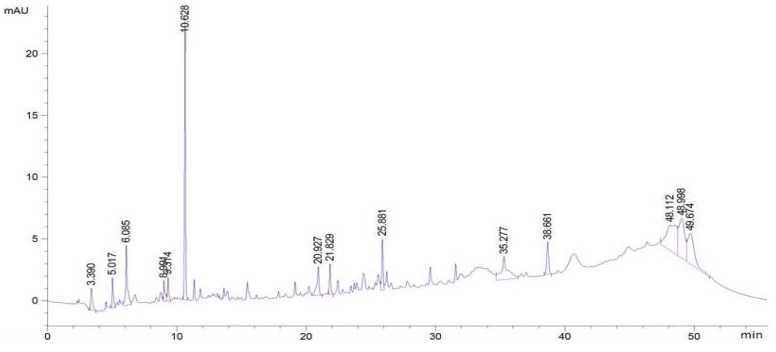
High performance liquid chromatography (HPLC) chromatograms of phenolic extracts from the leaves of *H. zenkeri* recorded at 280 nm (TR: 19.10: 3,4-OH benzoic acid; 33.49: apigenin; 25.67: caffeic acid; 23.48: catechine; 29.43: eugenol; 14.38; gallic acid; 25.11: *O*-coumaric; 21.91:OH-tyrosol; 30.52: *P*-coumaric acid. 42.19: quercetin; 29.45: rutin; 25.55: syringic acid; 17.35: theobromine; 21.77: tyrosol and 25.27: vanillic acid).

**Figure 13:**
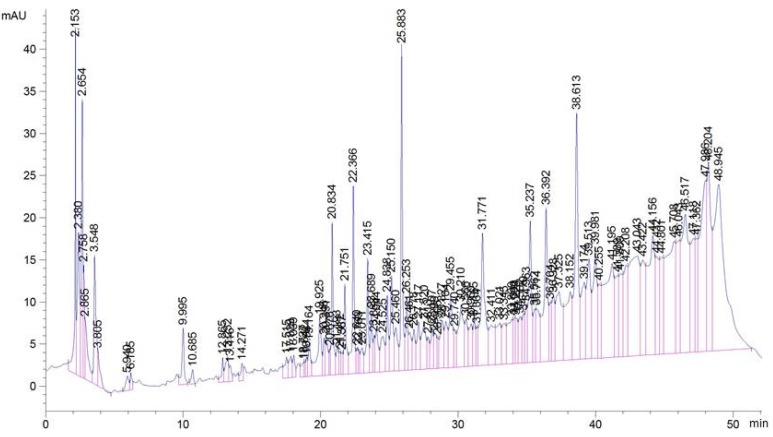
HPLC chromatograms of phenolic extracts from the barks of *H. zenkeri* recorded at 280 nm (TR: 19.10: 3,4-OH benzoic acid; 33.49:apigenin; 25.67: caffeic acid; 23.48: catechine; 29.43: eugenol; 14.38; gallic acid; 25.11: *O*-coumaric; 21.91:OH-tyrosol; 30.52: *P*-coumaric acid. 42.19: quercetin; 29.45: rutin; 25.55: syringic acid; 17.35: theobromine; 21.77: tyrosol and 25.27: vanillic acid.).

Various plants have been investigated to identify the presence of phenolic compounds using HPLC methods. The determination of phenolic compounds in the plant extracts helps to characterize them and to recognize their efficient uses as important plant resources [48]. The identification of phenolic compounds in extracts of *H. zenkeri* was carried out in this study and the phenolic compounds profile of the leaves and bark extracts are presented in Figure 12 and Figure 13 while the level of each identified phenol is shown in Table 2. The results demonstrated that the levels of phenolic molecules were higher in the bark than the leaves (Table 2). Three groups of polyphenols have been identified in the two samples. These include caffeic acid, OH-tyrosol acid, and Rutin, which were higher in the bark than in the leaves. The bark also possesses Quercetin, 3,4-OH benzoic acid, Eugenol, and Apigenin in higher concentrations than are found in the leaves. This result may explain the higher antioxidants potential observed in the bark samples of EEH and EEE compared to those of the leaves samples of EFH and EFE. However numerous pics that characterized other compounds have not been identified this could explain the activities observed in the leaves.

**Table 2:** Level of phenolic compounds in the different plant parts.

Standards Polyphenols *Characteristics*	*H. zenkeri* (Barks)		*H. zenkeri* (Leaves)	
	A (mUA)	Conc (mg/g DW)	A (mUA)	Conc (mg/g DW)
3,4-OH Benzoic Acid	46.54 ± 0.00	184 ± 0.00	0.00 ± 0.00	0.00 ± 0.00
Apigenin	96.31 ± 0.00	0.02 ± 0.00	0.00 ± 0.00	0.00 ± 0.00
Caffeic Acid	323.24 ± 0.00	6.06 ± 0.00	29.84 ± 0.00	0.56 ± 0.00
Catechine	132.8 ± 0.00	9.65 ± 0.00	0.00 ± 0.00	0.00 ± 0.00
Eugenol	52.9 ± 0.00	15.29 ± 0.00	0.00 ± 0.00	0.00 ± 0.00
Gallic Acid	25.15 ± 0.00	0.64 ± 0.00	0.00 ± 0.00	0.00 ± 0.00
*O*-Coumaric Acid	97.43 ± 0.00	3.01 ± 0.00	0.00 ± 0.00	0.00 ± 0.00
OH-Tyrosol	71.4 ± 0.00	6.60 ± 0.00	16.87 ± 0.00	1.56 ± 0.00
*P*-Coumaric acid	122.74 ± 0.00	2.40 ± 0.00	0.00 ± 0.00	0.00 ± 0.00
Quercetin	233.88 ± 0.00	21.97 ± 0.00	0.00 ± 0.00	0.00 ± 0.00
Rutin	131.5 ± 0.00	11.07 ± 0.00	22.9 ± 0.00	1.93 ± 0.00
Syringic Acid	85.5 ± 0.00	2.12 ± 0.00	0.00 ± 0.00	0.00 ± 0.00
Theobromine	94.73 ± 0.00	3.18 ± 0.00	0.00 ± 0.00	0.00 ± 0.00
Tyrosol	111.02 ± 0.00	6.49 ± 0.00	0.00 ± 0.00	0.00 ± 0.00
Vanillic Acid	148.93 ± 0.00	4.76 ± 0.00	0.00 ± 0.00	0.00 ± 0.00

Conc: concentration; DW: Dried Weight; A: area.

## 4. Conclusions

In conclusion, the ethanolic and hydro-ethanolic extract of the bark and leaves of *H. zenkeri* showed an antioxidant and protective potential against oxidative damage. The hydro-ethanolic extract of the bark EEH exhibited the most significantly elevated activities among the tested extracts. The HPLC based phenolic profile of the samples showed a higher amount of phenolic compounds in the bark than in the leaves. Nevertheless, additional experiments need to be conducted to purify and identify the active compounds as well as to elucidate the other biological effects on *in vivo* experimental models.

## Abbreviations

MDA: malonaldialdehyde
DPPH: 2,2-diphenyl-1-picrylhydrazyl
FRAP: ferric reducing ability of plasma
Vit C: Vitamine C
ABTS: 2,2-azinobis(3-ethylbenzthiazoline)-6-sulfonic acid
BHT: butylated hydroxyl toluene
ROS: reactive oxygen species
FRAP: Ferric reducing antioxidant power
MDA: malon-dialdehyde
H_2_O_2_: hydrogen peroxide
